# Progress in Multimodal Treatment for Advanced Esophageal Squamous Cell Carcinoma: Results of Multi-Institutional Trials Conducted in Japan

**DOI:** 10.3390/cancers13010051

**Published:** 2020-12-27

**Authors:** Kazuo Koyanagi, Kohei Kanamori, Yamato Ninomiya, Kentaro Yatabe, Tadashi Higuchi, Miho Yamamoto, Kohei Tajima, Soji Ozawa

**Affiliations:** Department of Gastroenterological Surgery, Tokai University School of Medicine, Isehara 259-1193, Japan; heyhey.cohey@gmail.com (K.K.); yamato.ninomiya@gmail.com (Y.N.); k-yatabe@tokai-u.jp (K.Y.); tadashi.h@tsc.u-tokai.ac.jp (T.H.); miho-n@is.icc.u-tokai.ac.jp (M.Y.); tadidas0203@gmail.com (K.T.); sozawa@tokai.ac.jp (S.O.)

**Keywords:** esophageal squamous cell carcinoma, multimodal treatment, neoadjuvant chemotherapy, neoadjuvant chemoradiotherapy, definitive chemoradiotherapy

## Abstract

**Simple Summary:**

In Japan, the therapeutic strategies for esophageal squamous cell carcinoma (ESCC) are based on the results of multi-institutional trials conducted by the Japan Esophageal Oncology Group (JEOG), a subgroup of the Japan Clinical Oncology Group (JCOG). Since there are several differences in the factors influencing the treatment approach for esophageal cancer between Eastern and Western countries, the therapeutic strategies adopted in Asian countries, especially Japan, are often different from those in Western countries. Because a transthoracic esophagectomy with three-field lymph node dissection has been performed as a standard surgical procedure for advanced thoracic ESCC in Japan, multimodal treatment for ESCC has been developed to improve the surgical outcomes after this relatively invasive surgical procedure. In this review, we describe the history and current status of therapeutic strategies for ESCC in Japan with a focus on the results of clinical trials conducted by the JEOG.

**Abstract:**

In Japan, the therapeutic strategies adopted for esophageal carcinoma are based on the results of multi-institutional trials conducted by the Japan Esophageal Oncology Group (JEOG), a subgroup of the Japan Clinical Oncology Group (JCOG). Owing to the differences in the proportion of patients with squamous cell carcinoma among all patients with esophageal carcinoma, chemotherapeutic drugs available, and surgical procedures employed, the therapeutic strategies adopted in Asian countries, especially Japan, are often different from those in Western countries. The emphasis in respect of postoperative adjuvant therapy for patients with advanced esophageal squamous cell carcinoma (ESCC) shifted from postoperative radiotherapy in the 1980s to postoperative chemotherapy in the 1990s. In the 2000s, the optimal timing of administration of perioperative adjuvant chemotherapy returned from the postoperative adjuvant setting to the preoperative neoadjuvant setting. Recently, the JEOG commenced a three-arm randomized controlled trial of neoadjuvant therapies (cisplatin + 5-fluorouracil (CF) vs. CF + docetaxel (DCF) vs. CF + radiation therapy (41.4 Gy) (CRT)) for localized advanced ESCC, and patient recruitment has been completed. Salvage and conversion surgeries for ESCC have been developed in Japan, and the JEOG has conducted phase I/II trials to confirm the feasibility and safety of such aggressive surgeries. At present, the JEOG is conducting several trials for patients with resectable and unresectable ESCC, according to the tumor stage. Herein, we present a review of the JEOG trials conducted for advanced ESCC.

## 1. Introduction

Multimodal treatment approaches have led to improved oncological outcomes of patients with esophageal squamous cell carcinoma (ESCC) worldwide [1]. Since there are several differences in the factors influencing the treatment approach for esophageal cancer, such as differences in the proportion of patients with squamous cell carcinoma among all patients with esophageal carcinoma, chemotherapeutic drugs available, and surgical procedures employed, between Eastern and Western countries, the therapeutic strategies adopted in Asian countries, especially Japan, are often different from those in Western countries [2]. In Japan, a transthoracic esophagectomy with cervical, mediastinal, and abdominal (three-field) lymph node dissection has been established and still performed as a standard surgical procedure for advanced thoracic ESCC [3]. Therefore, multimodal treatment for esophageal squamous cell carcinoma has been developed to improve the surgical outcomes after this relatively invasive surgical procedure. On the other hand, in many Western countries, the Ivor Louis esophagectomy is the standard perioperative treatment. In addition to the surgical procedures, the strategy of multimodal treatment for advanced ESCC, such as tumors invading deeper than the muscle layer and/or with regional lymph node metastasis, has evolved differently in Japan and in Western countries.

Considering these characteristics of surgical procedures in Japan, the Japan Esophageal Oncology Group (JEOG), a subgroup of the Japan Clinical Oncology Group (JCOG) has developed the optimal therapeutic strategies for advanced ESCC that were determined by multi-institutional trials [4]. We describe the history and current status of therapeutic strategies for esophageal squamous cell carcinoma in Japan and also the differences to those of Western countries, with a focus on the results of clinical trials conducted by the JEOG.

## 2. Postoperative and Preoperative Therapies Used for ESCC in Japan

### 2.1. History of Postoperative Therapy

#### 2.1.1. Preoperative and Postoperative Radiotherapy

Preoperative radiation therapy was the predominantly employed postoperative treatment for ESCC in the 1970s. It was generally believed that this approach would improve the resectability of the primary tumor and prevent local tumor recurrence [5]. On the other hand, the superiority of postoperative radiotherapy was highlighted by some research groups, who reported lower postoperative morbidity and improved survival rates, based on a retrospective comparison with a control group [6]. Therefore, the JEOG conducted a randomized controlled trial (RCT) to determine which mode of radiotherapy, preoperative or postoperative radiotherapy, would provide better survival. This study (JCOG 8201, 1981–1983) compared preoperative radiotherapy (30 Gy) plus postoperative radiotherapy (24 Gy) with postoperative radiotherapy (50 Gy) only (Table 1). The results revealed significantly better survival rates in the surgery plus postoperative radiotherapy alone group as compared to that in the surgery plus preoperative radiotherapy plus postoperative radiotherapy group [7]. The results prompted a switch of the timing of administration of postoperative therapy for ESCC from preoperative to postoperative, and there was a general movement away from preoperative radiotherapy to postoperative radiotherapy.

#### 2.1.2. Postoperative Radiotherapy versus Postoperative Chemotherapy

In Japan, cisplatin has been considered a useful drug for the treatment of esophageal cancer since the early 1980s. The JEOG conducted a RCT to determine whether postoperative radiation therapy or postoperative chemotherapy might provide the better survival rate. This study (JCOG8503, 1984–1987) compared postoperative radiotherapy (50 Gy) with postoperative chemotherapy (cisplatin 70 mg/m^2^ + vindesine 3 mg/m^2^ × 2 courses). At the time of this trial, 5-FU was not yet widely available, and the combination of cisplatin plus vindesine, which was the standard treatment for non-small-cell lung cancer at that time, was used in the study. Although the study did not reveal any significant difference in the 5-year overall survival (OS) between the two treatment arms, the results at least suggested that postoperative chemotherapy was not inferior to postoperative radiotherapy, which was the standard of care in the world at the time [8]. Therefore, chemotherapy with cisplatin became generally accepted as the postoperative treatment regimen for ESCC in Japan.

#### 2.1.3. Postoperative Chemotherapy versus Surgery Alone

There have been marked advances in the surgical techniques used for esophageal cancer surgery, especially in regard to lymphadenectomy, including specific resection of the superior mediastinal cervical lymph nodes, which has become a standard procedure in Japan since the late 1980s. Therefore, the JEOG conducted a RCT to investigate whether postoperative chemotherapy could improve the survival in patients undergoing esophagectomy with three-field lymphadenectomy. This study (JCOG 8806, 1988–1991) compared surgery alone with surgery plus postoperative chemotherapy (cisplatin 70 mg/m^2^ + vindesine 3 mg/m^2^ × 2 courses) and revealed no significant difference in the 5-year OS rate between the two groups, so that esophagectomy with three-field lymphadenectomy alone was adopted as the standard of care for ESCC at that time [9].

Thereafter, two phase II trials indicated that CF was superior to combined cisplatin plus vindesine as postoperative therapy for advanced esophageal cancer. Therefore, the JEOG initiated a RCT to determine whether postoperative chemotherapy with CF was additively effective in improving survival as compared to surgery with two- or three-field lymphadenectomy alone for ESCC, pathologic stage II/III, except T4. This trial (JCOG 9204, 1992–1997) compared surgery alone with surgery plus postoperative chemotherapy (80 mg/m^2^ cisplatin on day 1 + 800 mg/m^2^ 5-FU on days 1–5 × 2 courses). The primary endpoint of the study was the 5-year disease-free survival (DFS) rate, and the DFS rate in the postoperative chemotherapy group (120 patients) was better than that in the surgery-alone group (122 patients) (55% vs. 45%, *p* = 0.04); the 5-year OS rates were 61% and 52% (*p* = 0.13), respectively. The improved outcomes with postoperative chemotherapy were more pronounced in the subgroup with lymph node metastases [10]. Based on these findings, surgery followed by postoperative CF chemotherapy was considered as the standard of care for advanced ESCC patients in the late 1990s.

#### 2.1.4. Postoperative Chemotherapy versus Preoperative Chemotherapy

While postoperative chemotherapy had been the standard of care for esophageal cancer in Japan, in the West, preoperative chemotherapy had remained the mainstay of treatment due to the high invasiveness of and morbidity associated with surgery [11]. Therefore, it remained controversial as to whether preoperative chemotherapy might have a superior effect on the survival of esophageal cancer patients as compared to surgery alone or surgery plus postoperative chemotherapy. Therefore, the JEOG conducted a RCT to determine the optimal timing of chemotherapy (preoperative vs. postoperative) in patients with locally advanced ESCC. In this study (JCOG9907, 2000–2006), patients with clinical stage II/III ESCC, excluding T4 cases, were randomly assigned to groups that received either preoperative or postoperative chemotherapy (cisplatin 80 mg/m^2^ on day 1 and 5-FU 800 mg/m^2^ continuous infusion over days 1–5, up to 2 courses with a 3-week interval). The primary endpoint of progression-free survival did not reach the discontinuation boundary, but the OS was better in the preoperative chemotherapy group (164 patients) than in the postoperative chemotherapy group (166 patients) (*p* = 0.01). An updated analysis showed that the 5-year OS rate was 43% in the postoperative chemotherapy group and 55% in the preoperative chemotherapy group (hazard ratio (HR), 0.73, 95% confidence interval (CI), 0.54–0.99, *p* = 0.04) [12]. In addition, preoperative chemotherapy was not associated with an increased risk of postoperative complications or hospital mortality [13].

**Figure 1:**
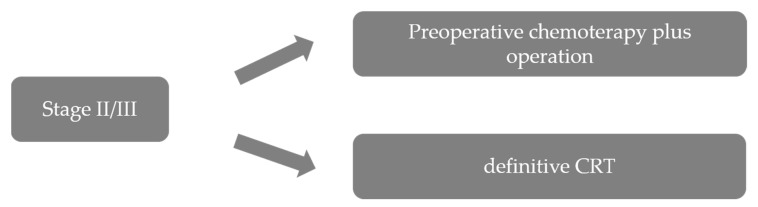
Treatment strategies for stage II/III ESCC, adapted and modified from guidelines of the Japan Esophageal Society. ESCC, esophageal squamous cell carcinoma; CRT, chemoradiotherapy.

There were three possible reasons for the favorable results of preoperative chemotherapy in this trial. First, downstaging was achieved with preoperative chemotherapy in some patients. The proportion of patients with clinical stage II was similar in the two groups, but the proportion of patients with pathological stage II or less was higher in the preoperative than in the postoperative chemotherapy group. Second, the frequency of complete resection (R0) was slightly higher in the preoperative chemotherapy group than in the postoperative chemotherapy group. Third, the completion rate of the protocol treatment was much higher in the preoperative chemotherapy group than in the postoperative chemotherapy group; protocol-based treatment with two courses of chemotherapy and R0 resection was completed in 85.4% of patients in the preoperative chemotherapy group, but in only 75.0% of patients of the postoperative chemotherapy group [14]. These results led to preoperative chemotherapy with CF becoming established as the standard of care for patients with stage II/III ESCC in Japan (Figure 1). Thus, the optimal timing of chemotherapy again changed from postoperative chemotherapy to preoperative chemotherapy.

#### 2.1.5. Postoperative Chemotherapy for ESCC in Western Countries

Reports of postoperative chemotherapy for ESCC were very few. The French Association for Surgical Research performed a randomized controlled trial comparing surgery alone with postoperative chemotherapy using CF for patients with ESSC [15] (Table 2). Before randomization, they separated 120 patients into two groups, curative complete resection and palliative resection leaving residual macroscopic or microscopic tumor tissue. As a result, OS was similar in the two groups, with almost identical medians of 13 months in the postoperative group and 14 months in the surgery-alone group. The survival curves with or without chemotherapy were similar in the curative resection group and also in the palliative resection group. Based on these data, it was concluded that CF followed by surgery is not useful for patients with ESCC.

#### 2.1.6. Preoperative Chemotherapy for ESCC in Western Countries

Ancona and colleagues compared surgery alone with preoperative chemotherapy using CF plus surgery for stage II/III ESCC [16]. The surgical procedure adopted in this study was transthoracic esophagectomy combined with two-field lymphadenectomy. The 5-year OS (primary endpoint) was 22% in the surgery-alone group and 34% in the preoperative chemotherapy group (*p* = 0.55). They concluded that improved long-term survival was obtained in patients with clinically resectable ESCC who underwent preoperative chemotherapy and obtained a pathologic complete response. They also emphasized the necessity of major efforts to identify patients who are likely to respond to preoperative chemotherapy.

Two pivotal RCTs in terms of preoperative chemotherapy are known worldwide, the Radiation Therapy Oncology Group (RTOG) and the Medical Research Council (MRC) trials, although both squamous cell carcinoma and adenocarcinoma were included. The RTOG trial compared surgery alone with preoperative chemotherapy using CF plus surgery followed by two courses of postoperative chemotherapy in operable esophageal cancer cases [17]. More than 50% of patients (53% in the surgery-alone group and 54% in the preoperative chemotherapy group) consisted of adenocarcinoma, and both transthoracic and transhiatal esophagectomy were performed as the surgical procedures without limiting the extent of lymphadenectomy. The median survival was 16.1 months in the surgery-alone group and 14.9 months in the preoperative chemotherapy group (*p* = 0.53). There were no differences in survival between patients with squamous cell carcinoma and those with adenocarcinoma. They concluded that preoperative chemotherapy with a combination of CF did not improve OS among patients with squamous cell carcinoma or adenocarcinoma. They reported, in a long-term update, that the median survival times were 1.3 years for patients receiving preoperative chemotherapy versus 1.3 years for those undergoing surgery alone [18]. They described similar outcomes to those described by other researchers, with objective response to preoperative chemotherapy being associated with better survival. Investigators in the Medical Research Council Oesophageal Cancer Working Party compared surgery alone with preoperative chemotherapy using CF plus surgery for resectable esophageal cancer [19]. Two-thirds of patients (67% in the surgery-alone group and 66% in the preoperative chemotherapy group) consisted of adenocarcinoma, and the surgical procedure was chosen by the operating surgeon. The median survival was 13.3 months in the surgery-alone group and 16.8 months in the preoperative chemotherapy group (*p* = 0.004), and the 2-year survival rates were 34% and 43%, respectively. Hazard ratios for treatment effect in patients with squamous cell carcinoma and those with adenocarcinoma were the same, showing that the effects of treatment were extremely similar for both histologic types. They concluded that preoperative chemotherapy improved survival in patients with resectable esophageal cancer. In long-term update results of this trial, they reported that the 5-year survival was 17.1% in the surgery-alone group and 23.0% in the preoperative chemotherapy group, with consistent treatment effect achieved in both histologic types [20]. They emphasized that preoperative chemotherapy is an essential standard of care for patients with resectable esophageal cancer. Because two pivotal studies demonstrated completely different conclusions, the benefit of preoperative chemotherapy, even when limited to patients with ESCC was controversial, and there seems to be no current worldwide consensus as to the optimal preoperative chemotherapy.

#### 2.1.7. Preoperative Chemoradiotherapy for ESCC in Western Countries

A Dutch group (Chemoradiotherapy for Oesophageal Cancer Followed by Surgery Study (CROSS) Group) compared surgery alone with preoperative chemoradiotherapy followed by surgery for potentially curable squamous cell carcinoma (23%) or adenocarcinoma (75%) of the esophagus or the esophagogastric junction. A transthoracic esophagectomy with two-field lymphadenectomy was performed. A transhiatal resection was preferred for the tumors involving the esophagogastric junction. The median survival was 48.6 months in the preoperative chemoradiotherapy group and 24.0 months in the surgery-alone group (*p* = 0.003), and 81.6 months and 21.1 months, respectively, for patients with squamous cell carcinoma. The 5-year OS was 47% and 33%, respectively. They concluded that preoperative chemoradiotherapy improved survival among patients with potentially curable esophageal or esophagogastric junction cancer, regardless of histologic subtype [21]. The result of this study supported preoperative chemoradiotherapy as a standard of care for locally advanced esophageal cancer in Western countries in which adenocarcinoma is predominant histologic type.

The most recent meta-analysis by Sjoquist et al. [22] included 12 RCTs comparing preoperative chemoradiotherapy vs. surgery alone, with a total of 1854 patients. A significant survival benefit was evident for preoperative chemoradiotherapy with an HR of 0.78 (0.70–0.88; *p* < 0.0001). In a subgroup analysis, the HR for squamous cell carcinoma was 0.80 (0.68–0.93; *p* = 0.004) and for adenocarcinoma it was 0.75 (0.59–0.95; *p* = 0.02). This updated meta-analysis provided stronger evidence for a survival benefit than the former meta-analysis conducted by the same group [23]. This analysis also compared preoperative chemotherapy to preoperative chemoradiotherapy and demonstrated a non-statistically significant survival benefit for preoperative chemoradiotherapy (HR 0.88, 0.76–1.01; *p* = 0.07). Therefore, controversy still exists as to whether preoperative chemotherapy or preoperative chemoradiotherapy is more beneficial [24]. A RCT comparing preoperative chemotherapy using CF (91 patients including 25 SCC patients) with preoperative chemoradiotherapy (90 patients including 25 SCC patients) was conducted in Sweden and Norway. They revealed that the addition of radiotherapy to preoperative chemotherapy resulted in higher pathological complete response (pCR) rate and higher R0 resection rate, without significantly affecting survival [25].

### 2.2. Future Candidates for Preoperative Therapy for ESCC

#### 2.2.1. Adequate Preoperative Therapy

The results of a subgroup analysis in the JCOG9907 study showed that preoperative chemotherapy was more effective in clinical stage II or T1–2 esophageal cancer patients than in stage III or T3 patients, i.e., in patients with relatively early-stage disease. Furthermore, the low single-site recurrence rates of 31% and 25% in cases of tumor recurrence in the two groups could be attributable to our elaborate surgical technique. The results of this study suggested that preoperative chemotherapy with cisplatin plus 5-FU would be a good treatment strategy when aggressive surgery provides adequate local tumor control, but when the local tumor control is inadequate, more aggressive adjuvant therapy, such as more intensive preoperative chemotherapy or preoperative chemoradiotherapy, may be the preferable treatment strategy to obtain adequate local tumor control as well as systemic disease control. Docetaxel is one of the most promising agents for esophageal cancer, and an exploratory study of preoperative chemotherapy with DCF for locally advanced ESCC showed a favorable response rate (61.5%), with no treatment-related deaths. The therapeutic promise of docetaxel was demonstrated in a randomized phase II trial [26]. However, the clinical question of whether preoperative chemotherapy or preoperative chemoradiotherapy is superior still remained unresolved.

Under this circumstance, the JEOG initiated a three-arm randomized controlled trial (JCOG1109) in 2012, to confirm the superiority of DCF and chemoradiotherapy with CF (CF-RT) over CF as preoperative therapy for locally advanced ESCC in terms of the OS [27]. Patients in group A were treated with two courses of preoperative CF (cisplatin 80 mg/m^2^ on day 1, 5-FU 800 mg/m^2^ on days 1–5) repeated every three weeks; group B received three courses of preoperative DCF (70 mg/m^2^ docetaxel on day 1, 70 mg/m^2^ cisplatin on day 1, 750 mg/m^2^ 5-FU on days 1–5) repeated every three weeks; group C received two courses of preoperative chemoradiation (41.4 Gy/23 fractions) with two courses of CF (75 mg/m^2^ cisplatin on day 1 plus 5-FU 1000 mg/m^2^ on days 1–4) repeated every four weeks. Both transthoracic open esophagectomy and minimally invasive esophagectomy were acceptable in all three groups, and surgery should be performed within 56 days after completion of the preoperative treatment. Patient enrollment was completed in 2018 and follow-up of the enrolled patients is ongoing.

#### 2.2.2. Preoperative Therapy with Immune Checkpoint Inhibitors

Nivolumab and pembrolizumab are immune checkpoint inhibitors, newly developed drugs with antitumor activity. Until recently, no molecular-targeted drugs were approved for the treatment of advanced esophageal cancer, but in 2019, the FDA approved pembrolizumab as a second- or subsequent-line treatment for PD-L1-positive cases [28]. Strong PD-L1 expression is generally observed in esophageal cancers, with reported expression levels in the tumor cells of 15–83% and in immune cells of 13–31% [28,29,30,31]. Furthermore, in 2019, an international phase III trial (ATTRACTION-3) showed that nivolumab significantly prolonged the OS in patients with unresectable advanced or recurrent esophageal cancer who were refractory or intolerant to fluoropyrimidine and platinum as compared to the existing taxanes in an international phase III trial [32]. Thus, in Japan, nivolumab was approved in 2020 for use as second-line chemotherapy for patients with unresectable advanced or recurrent esophageal cancer. Based on these results, the JEOG has initiated a phase I trial (JCOG1804E) to evaluate the efficacy of preoperative chemotherapy with the addition of nivolumab to CF and DCF [33].

## 3. Surgery for ESCC in Japan

### 3.1. Techniques of Esohagectomy in Japan

#### 3.1.1. Approach to Esophagectomy

Open thoracic and abdominal surgery remained the only surgical strategies adopted for esophageal cancer, before Cuschieri first reported thoracoscopic esophagectomy in 1992 [34]. Since then, thoracoscopic/laparoscopic surgery has been developed rapidly and is now considered as a less-invasive approach than open surgery. To date, several thoracoscopic or laparoscopic approaches for resection of thoracic esophageal cancer have come to be defined as minimally invasive esophagectomy (MIE), based on the tumor location, clinical stage, and patient demographics [35,36]. Although, total thoracoscopic esophagectomy and laparoscopic esophagectomy are representative of (total) MIE in a narrow sense, video-assisted thoracoscopic surgery (VATS) [37], esophagectomy with mini thoracotomy (up to an approximately 5-cm incision) in a wider sense, and laparoscopic approaches are also considered as falling within the scope of MIE [38]. In Japan, Akaishi et al. were the first to report the use of thoracoscopic total esophagectomy with en bloc mediastinal lymphadenectomy in 1996 [39]. After these exploratory studies, the number of MIEs performed has increased and there have been advances towards the standardization of surgical techniques.

We reviewed studies that compared the surgical outcomes of open transthoracic esophagectomy (OE) and MIE and found that MIE was not inferior to OE in terms of the accuracy of lymph node dissection and surgical invasiveness, and might also be associated with a reduced risk of respiratory complications [40].

Robot-assisted MIE (RAMIE) was first described in 2004 by Kernstine et al. [41]. In 2006, they published an account of their first experience with the use of RAMIE in combination with conventional laparoscopic surgery and showed that this new surgical procedure is technically feasible and associated with lower blood loss [42].

The da Vinci Robotic Systems (Intuitive, Sunnyvale, CA, USA) provides a three-dimensional magnified view of the surgical field [43]. Because of the theoretical advantages of robot-assisted surgery, including articulation of the instruments, tremor filtering, features allowing minimization of large movements for the surgeon, and better ergonomics, it has the potential to accelerate the MIE learning curve. The increased degree of freedom provided by the articulation of the surgical instruments might overcome the movement restrictions caused by the rib cage and improve the accuracy of lymph node dissection around the recurrent laryngeal nerves [44], which could be expected to lead to better outcomes and avoidance of recurrent laryngeal paralysis. Although RAMIE is spreading rapidly in Japan based on these theoretical advantages, there are still several issues to be investigated—the expensive cost of da Vinci Robotic Systems and the clinical usefulness compared with thoracoscopic surgery.

#### 3.1.2. Three-Field Lymph Nodes Dissection

The importance of lymphadenectomy in esophageal cancer surgery has been well established around the world. Ever since Torek reported the first case of successful esophagectomy in 1913 [45], the safety and efficacy of esophagectomy for esophageal cancer have been reported by numerous researchers, and the range of lymph node (LN) dissection has been extended. Three-field LN dissection (3FD) was initiated by two Japanese Surgeons. Kajitani was the first to perform systematic dissection of the LNs around the recurrent laryngeal nerves and developed upper mediastinal LN dissection [46]. Sannohe then reported cervical LN dissection and the incidence of metastases in patients who underwent 3FD [47]. Following these reports, the safety and survival rates of 3FD were also shown by a number of reports in Japan [48,49,50,51]. Initially, surgical procedures were performed without considering recurrent laryngeal nerves, and recurrent laryngeal nerve paralysis occurred in many patients. However, with the improvement in surgical technique around recurrent laryngeal nerves, the frequency of recurrent laryngeal nerve paralysis had gradually decreased. In the 1990’s, 3FD was accepted worldwide and its safety was established [52,53,54]. Kato et al. reported that patients with esophageal cancer who underwent 3FD showed better OS rates than those who received 2FD [55]. Igaki et al. showed that neck dissection is also important in patients with lower thoracic ESCC. They reported that 3FD for patients with LN metastases in the upper and/or central mediastinum can improve the survival rate as compared to 2FD, even in patients with lower thoracic ESCC [3]. Moreover, Altorki et al. reported 80 patients who underwent esophagectomy with 3FD, in 30% of whom the disease was upstaged following 3FD [52]. Based on these previous reports, esophagectomy with 3FD is currently adopted as the standard procedure for thoracic esophageal cancer in Japan.

We speculate the following reasons for the prolonged postoperative survival associated with 3FD. First, extended LN dissections may increase the curative potential of esophagectomy. Esophageal cancer can cause LN metastasis extending from the cervical to the abdominal fields, and an extended range of LN dissection could be expected to lead to better elimination of tumor cells. Second, extended LN dissection improves the accuracy of staging and leads to better postoperative survival in patients with each disease stage. Even if an upper mediastinal LN dissection could be performed with both 3FD and 2FD, the number of dissected LNs in the upper mediastinum would be higher in 3FD, because of the added cervical approach in 3FD. Therefore, 3FD may be beneficial for obtaining better postoperative survival.

Recently, several systematic reviews and meta-analyses have been reported. Shang et al. analyzed the long-term survival of the patients and showed that 3FD was superior to 2FD in patients with LN metastasis in the cervical or upper mediastinal LNs [56]. Ma et al. also conducted a meta-analysis and reported that 3FD was associated with improved survival rates following esophagectomy [57].

### 3.2. Salvage and Conversion Surgery

#### 3.2.1. Terminology

**Figure 2:**
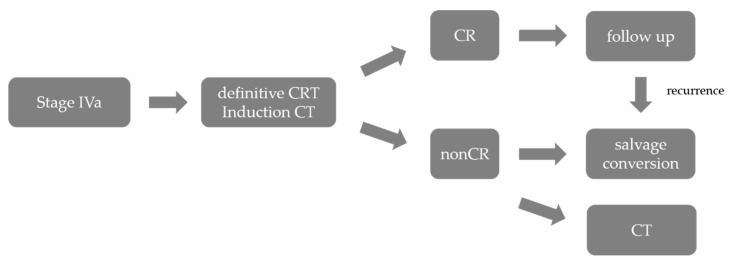
Treatment strategies for stage IVa ESCC, adapted and modified from guidelines of the Japan Esophageal Society. ESCC, esophageal squamous cell carcinoma; CRT, chemoradiotherapy; CT, chemotherapy; CR complete response.

Salvage surgery is generally considered as a surgical procedure for radical resection of a lesion that has failed to resolve, or that has resurfaced after radiation and/or chemotherapy, with the intent of cure. Conversion surgery, on the other hand, is considered to be a procedure that involves a change in treatment, such as from chemotherapy and/or radiation to surgical resection. The concept of induction chemotherapy, especially for patients with borderline resectable esophageal cancer, which involves deciding whether to perform conversion surgery or chemoradiotherapy after chemotherapy, has also been becoming popular recently, and clinical trials are under way in Japan. As described below, chemoradiation therapy for unresectable advanced esophageal cancer is also widely used. Salvage surgery and conversion surgery are playing increasingly important roles in the multimodal treatment of advanced esophageal cancer (Figure 2).

#### 3.2.2. Clinical Trials for Resectable ESCC

In 1999, a phase III trial conducted in the US in patients with T1-3N0-1M0 ESCC showed that CRT with CF concurrently with 50.4 Gy radiation yielded a significantly improved 5-year OS of 26%, as compared to 0% for radiation alone [58]. This made CRT a standard noninvasive treatment for patients who did not wish to undergo surgery. Therefore, the JEOG conducted a phase II study (JCOG9906, 2000–2002) to evaluate the efficacy and safety of CRT in patients with Stage II/III ESCC (Table 3). CRT was performed in 96 patients and the outcomes in the CRT arm were comparable to those in the preoperative chemotherapy plus surgery arm, and the toxicity was manageable. However, late toxicities, comprising Grade 3/4 esophagitis (13%), pericardial (16%) and pleural (9%) effusion, and radiation pneumonitis (4%), were observed, causing four deaths, and it was concluded that further improvement was required for reduction in the incidence of late toxicities [59].

In Japan, based on the results of trials, definitive CRT (dCRT) with 60 Gy of radiation in combination with CF therapy is adopted for patients with Stage II/III ESCC who do not wish to undergo surgery. However, the high incidence of late toxicities and of complications after salvage surgery have made dCRT for Stage II/III ESCC an option that could be considered. For this reason, the JEOG conducted a single-arm confirmatory study (JCOG 0909, 2010–2014) to evaluate the reduction in the incidence of late toxicities with a reduced radiation dose to 50.4 Gy, the improvement in the outcomes with the inclusion of salvage therapy, and the safety of salvage therapy. The 3-year OS rate was 74.2% (90% CI: 65.9–80.8), which was higher than the expected rate of 55%. Salvage surgery resulted in Grade 3/4 postoperative mobility in five patients (20%) and postoperative death in one patient (4%), but R0 surgery was possible in 76% of cases [60]. Salvage surgery was considered as an effective treatment option for limited indications, and CRT, consisting of radiotherapy at 50.4 Gy plus CF, became the standard of care for esophageal cancer patients in Japan who preferred nonsurgical treatment.

#### 3.2.3. Clinical Trials for Unresectable ESCC

The JEOG conducted a phase II/III trial (JCOG0303, 2004–2009) of CRT for esophageal cancer patients with T4b disease or unresectable lymph nodes with standard-dose CF (Arm A) versus low-dose CF (Arm B). Because there were no differences in the toxicities between the two arms, Arm B was judged as not worthy of further evaluation in the phase III setting and the study was terminated [61]. Daily RT plus low-dose CF chemotherapy did not qualify for further evaluation as a new treatment option for patients with locally advanced unresectable esophageal cancer.

In 2013, a multicenter phase II trial (COSMOS trial) was performed to assess the safety and efficacy of induction DCF chemotherapy and subsequent conversion surgery for initially unresectable locally advanced ESCC. Conversion surgery was performed in 41.7% of patients after induction chemotherapy or subsequent CRT, and R0 resection was achieved in 95% of these patients, with no serious postoperative complications. Induction DCF chemotherapy followed by conversion surgery as a multidisciplinary treatment strategy showed promise in terms of both the tolerability and efficacy in patients with locally advanced unresectable ESCC [62]. Based on these results, the JEOG designed a phase III trial (JCOG1510) to determine the outcomes of conversion surgery after induction chemotherapy [63]. The purpose of this study was to confirm the superiority, in terms of the OS, of induction chemotherapy with DCF followed by conversion surgery or dCRT over dCRT alone OS in patients with locally advanced unresectable ESCC, and patient enrollment is ongoing.

## 4. Discussion

In this review, we have shown the history and results of RCTs for advanced ESCC in Japan and also presented ongoing RCT conducted by JEOG. Thanks to the JEOG’s continued efforts, we have been able to achieve better outcomes for advanced ESCC. Rationale for multimodal treatment for advanced ESCC in Japan is different from other Western countries—multimodal treatment for ESCC has been shown to improve the outcomes after surgical treatment. Non-surgical treatment has not been considered as standard treatment, therefore, RCT that compared the superiority or non-inferiority of non-surgical treatment over the esophagectomy has not been conducted. There are two possible reasons for this. One is that the prognosis of the patients was dramatically improved after introduction of three-field lymph node dissection. The other is that few drugs have been approved by national insurance and used for ESCC in Japan. In addition to cisplatin and 5-FU, until 2019, only docetaxel and paclitaxel could be used. In 2020, nivolumab was approved and is now available for unresectable and/or recurrent ESCC. Given these backgrounds, we believe that new perspectives should be considered for multimodal treatment of advanced ESCC. Quality of life (QOL) is considered as an important issue. Although three-field lymph node dissection could contribute the better prognosis, this kind of invasive procedure might reduce the patient’s QOL. We also need to investigate new drugs that can be used for ESCC.

Identification of responders and non-responders for chemotherapy and radiotherapy is an urgent need based on the evidence that histological complete response is predictive of long overall survival. If it were possible to predict responders and non-responders, unnecessary toxicity, and time caused by unnecessary preoperative chemotherapy or chemoradiotherapy, could be avoided. Therefore, future RCT should focus on the identification of prognostic and predictive biomarkers as well as the integration of molecular targets. Clinical trials incorporating molecularly targeted therapeutics into multimodal treatment for esophageal cancer are being initiated. Although promising results have not been demonstrated yet, development of molecularly targeted drugs could contribute the progress of multimodal treatment for advanced ESCC.

Immune checkpoint inhibitors are already available in ESCC and are another promising candidate for multimodal treatment because strong PD-L1 expression is generally observed in esophageal cancers, with reported expression levels in the tumor cells of 15–83% and in immune cells of 13–31% [28,29,30,31]. Furthermore, in 2019, an international phase III trial (ATTRACTION-3) showed that nivolumab significantly prolonged the OS in patients with unresectable advanced or recurrent esophageal cancer who were refractory or intolerant to fluoropyrimidine and platinum as compared to the existing taxanes in an international phase III trial [32]. Immune checkpoint inhibitors have a different mechanism of action in cancer tissues to that of traditional anticancer drugs. Thus, nivolumab as well as well as pembrolizumab are expected to be studied as very promising candidates for multimodal treatment for advanced ESCC.

## 5. Conclusions

We have summarized the history and current status of multidisciplinary treatment for ESCC in Japan, mainly based on the results of clinical trials conducted by the JEOG. We are expecting the results of a three-arm randomized controlled trial of preoperative chemotherapy (JCOG1109) and a phase I trial (JCOG1804E) to evaluate the efficacy of adding nivolumab to preoperative CF and DCF.

## Figures and Tables

**Table 1:** Overview of clinical trials of adjuvant therapy for ESCC in Japan.

Trial	Year	Stage Enrollment ^†^	Phase	Group	*n*	Primary Endpoint	*p* Value	Summary
JCOG8201	1981–1983	I–III	III	Preoperative + postoperative RT	104	OS *: 13.1	<0.01	Postoperative RT alone group was superior.
				Postoperative RT alone	103	OS *: 21.6		
JCOG8503	1984–1987	I–IV (resectable)	III	Postoperative RT	127	5-year OS: 44%	N.S.	
				Postoperative CT (CV)	126	5-year OS: 42%		
JCOG8806	1988–1991	I–IV (resectable)	III	Surgery alone	100	5-year OS: 44.9%	N.S.	
				Surgery + postoperative CT (CV)	105	5-year OS: 48.1%		
JCOG9204	1992–1997	II/III, excluding T4	III	Surgery alone	122	5-year DFS: 45%	0.04	Surgery plus postoperative CT group was superior.
				Surgery + postoperative CT (CF)	120	5-year DFS: 55%		
JCOG9907	2000–2006	II/III, excluding T4	III	Preoperative CT (CF)	164	5-year OS: 55%	0.04	Preoperative CT group was superior.
				Postoperative CT (CF)	161	5-year OS: 43%		
JCOG1109	2012–2018	II/III, excluding T4	III	Preoperative CT (CF)		OS		Follow-up is ongoing.
				Preoperative CT (DCF)				
				Preoperative CRT				

ESCC, esophageal squamous cell carcinoma; RT, radiotherapy; OS, overall survival; CT, chemotherapy; CV, cisplatin plus vindesine; N.S., not significant; DFS, disease-free survival; CF, cisplatin + 5-FU; DCF, docetaxel, cisplatin plus 5-FU; CRT, chemoradiotherapy; ^†^ UICC at the time; * median, month.

**Table 2:** Overview of clinical trials of adjuvant therapy for ESCC in Western countries.

Authors	Year	Stage Enrollment ^†^	Phase	Group	*n*	Primary Endpoint	*p* Value	Summary
Pouliquen [15]	1987–1992	II–IV	III	Surgery alone	68	OS *: 14	N.S.	CF followed by surgery is not useful.
				Preoperative CT (CF)	52	OS *: 13		
Ancona [16]	1992–1997	II/III	III	Surgery alone	47	5-year OS: 22%	N.S.	OS was improved only in the patients with pCR.
				Surgery + postoperative CT (CF)	47	5-year OS: 34%		
Kelsen [17]	1990–1995	I/II/III	III	Surgery + postoperative CT (CF)	100	OS *: 16.1	N.S.	Preoperative chemotherapy with CF did not improve OS.
(RTOG trial)		(AC: 53%)		Surgery + pre and postoperative CT (CF)	105	OS *: 14.9		
Working group ^‡^ [19]	1992–1998	I/II/III	III	Surgery alone	402	OS *: 13.3	<0.01	Preoperative CT improved survival.
(MRC trial)		(AC: 67%)		Surgery + preoperative CT (CF)	400	OS *: 16.8		
Shapiro [21]	2000–2004	I/II/III	III	Surgery alone	188	OS *: 81.6	<0.01	Preoperative CRT was effective.
		(AC: 75%)		Preoperative CRT	180	OS *: 21.1		
Sjoquist [22]			meta-analysis	Surgery alone	952	OS		Strong evidence for a benefit of preoperative CRT.
				Preoperative CRT	980	HR: 0.78 (0.70–0.88) §		
				Preoperative CT	1141	OS		Advantage of preoperative CRT was unclear.
				Preoperative CRT	1079	HR: 0.88 (0.76–1.01) §		
Klevebro [25]	2006–2013	I/II/III	III	Preoperative CT	91	pCR rate: 9%	<0.01	Preoperative CRT had higher pCR rate.
		(AC: 72%)		Preoperative CRT	90	pCR rate: 28%		

ESCC, esophageal squamous cell carcinoma; CT, chemotherapy; CF, cisplatin plus 5-FU; OS, overall survival; N.S., not significant; pCR, pathological complete response; AC, adenocarcinoma; CRT, chemoradiotherapy; ^†^ UICC at the time; * median, month; ^‡^ Medical Research Council Oesophageal Cancer Working Party; § 95% confidence interval.

**Table 3:** Summary of clinical trials of CRT or induction chemotherapy for ESCC conducted in Japan.

Trial	Year	Stage Enrollment ^†^	Phase	Group	*n*	Primary Endpoint	*p* Value	Summary
JCOG9906	2000–2002	II/III, excluding T4	II	CRT	76	OS *: 29		CRT was effective.
JCOG0909	2010–2014	II/III, excluding T4	II	CRT with/without salvage	94	3-year OS: 74.2%		CRT with salvage was effective and safe.
JCOG0303	2004–2009	T4b or unresectable LN	II	CRT (standard-dose CF)	71	OS *: 14.4	N.S.	Low-dose group was slightly inferior.
				CRT (low-dose CF)	71	OS *: 13.1		
COSMOS	2013–2014	T4b or unresectable LN	II	indDCF and CS/CRT	48	1-year OS: 67.9%		IndDCF and CS showed tolerability and efficacy.
JCOG1510	2016	T4b or unresectable LN	III	CRT		OS		Patient enrollment is ongoing.
	-			indDCF and CS/CRT				

CRT, chemoradiotherapy; ESCC, esophageal squamous cell carcinoma; OS, overall survival; CF, cisplatin plus 5-fluorouracil; LN, lymph node; N.S., not significant; indDCF, induction therapy with docetaxel, cisplatin, and 5-fulorouracil; CS, conversion surgery; ^†^ UICC at the time; * median, month.

## Data Availability

The data presented in this study are openly available.
